# Contribution of microreactor technology and flow chemistry to the development of green and sustainable synthesis

**DOI:** 10.3762/bjoc.13.51

**Published:** 2017-03-14

**Authors:** Flavio Fanelli, Giovanna Parisi, Leonardo Degennaro, Renzo Luisi

**Affiliations:** 1 Department of Pharmacy – Drug Sciences, University of Bari “A. Moro”, FLAME-Lab – Flow Chemistry and Microreactor Technology Laboratory, Via E. Orabona 4, 70125, Bari. Italy

**Keywords:** flash chemistry, flow chemistry, green chemistry, microreactor technology, sustainable synthesis

## Abstract

Microreactor technology and flow chemistry could play an important role in the development of green and sustainable synthetic processes. In this review, some recent relevant examples in the field of flash chemistry, catalysis, hazardous chemistry and continuous flow processing are described. Selected examples highlight the role that flow chemistry could play in the near future for a sustainable development.

## Introduction

**Figure 1:**
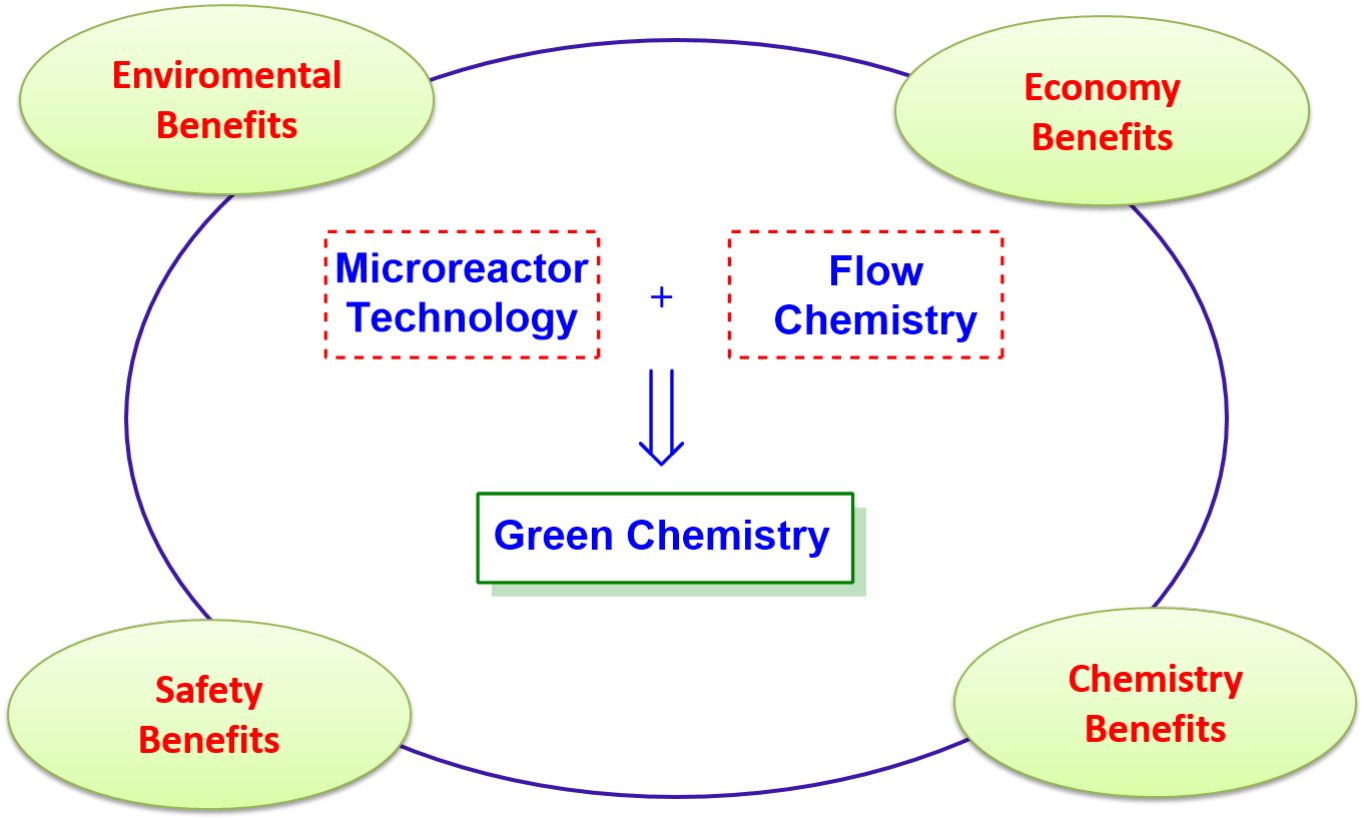
Microreactor technologies and flow chemistry for a sustainable chemistry.

Green chemistry’s birth was driven by the necessity to consider and face the urgent question of sustainability. Chemical production concerns an extended range of fields such as textiles, construction, food, cosmetic components, pharmaceuticals and so forth. An innovative approach to the chemistry world requires new strategies and criteria for an intelligent chemistry. It is understood that all this matter has big implications in economy and politics. Recent studies predicted a growth of green chemical processing up to $100 billion in 2020 (Pike Research study) [1]. All this offers important and arduous challenges expressed in terms of new synthetic strategies using sustainable, safe, and less toxic materials. On green chemistry we can read Paul Anastas and John Warne’s 12 principles, set up in 1998, which illustrate the characteristics of a greener chemical process or product [2]. Microreactor technology and flow chemistry could play a pivotal role in the context of sustainable development. In fact, flow chemistry is becoming a new technique for fulfilling several of the twelve green chemistry principles. The microreactor approach, could provide protection, preserves atom economy, guarantees less hazardous chemical synthesis and allows the use of safer solvents and auxiliaries. Furthermore, it pushes towards designing of chemistry with a lower environmental and economic impact, enhance the importance of catalysis, allows real-time analysis for pollution prevention and provides inherently safer chemistry (Figure 1) [3]. Without claiming to be exhaustive, in this review we report recently published representative synthetic applications that demonstrate the growing contribution of flow chemistry and microreactor technology in green and sustainable synthesis [4–7].

## Review

### Flow microreactors: main features

The peculiar properties of microreactors [8] derive from their small size and can be ascribed mainly to the following characteristics: a) fast mixing: in a flow microreactor, in striking contrast to batch conditions, mixing takes place by molecular diffusion so that a concentration gradient can be avoided; b) high surface-to-volume ratio: the microstructure of microreactors allows for a very rapid heat transfer enabling fast cooling, heating and, hence, precise temperature control; c) residence time: it is the period of time the solution of reactants spend inside the reactor, and it gives a measure of the reaction time. The residence time is strictly dependent on the characteristics of the reactor (i.e., length of the channels, volume), and on the flow rate. The residence time is one of the crucial factors to be considered in optimizing flow reactions, especially when unstable or short-lived reactive intermediates are concerned. Microreactor technology provides also several benefits. Safety benefits, because of the high efficiency in heat exchange, and avoided accumulation of unstable intermediates. Economy benefits, due to lower manufacturing and operating costs, reduced work-up procedures, use of less raw materials and solvents and reduced waste. Chemistry benefits associated to the use of microreactor technology are the improved yields and selectivities, the possibility to conduct reactions difficult or even impossible to perform in batch, and the use of reaction conditions that allow exploring new chemical windows [9].

### Contribution of flash chemistry to green and sustainable synthesis

**Scheme 1:**
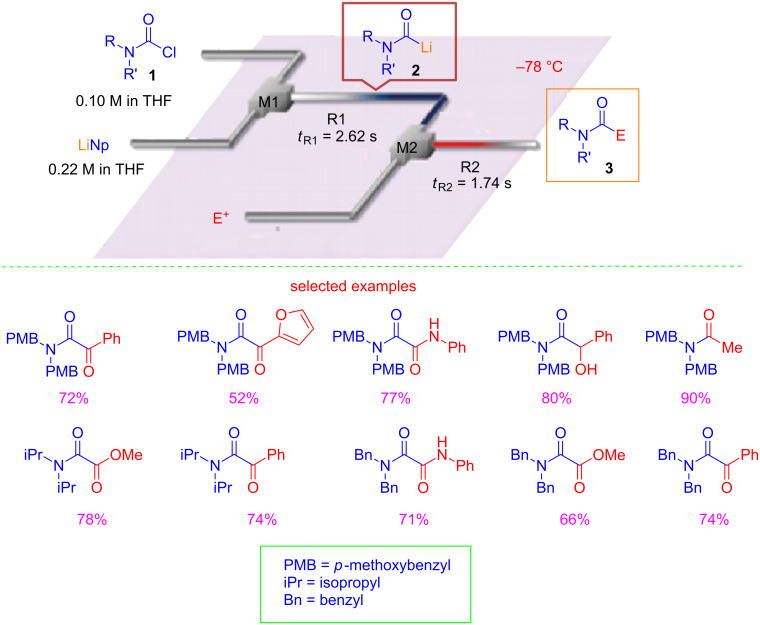
A flow microreactor system for the generation and trapping of highly unstable carbamoyllithium species.

The concept of flash chemistry as a "*field of chemical synthesis using flow microreactors where extremely fast reactions are conducted in a highly controlled manner to produce desired compounds with high selectivity*" was firstly introduced by Yoshida [10]. Flash chemistry can be considered a new concept in both organic and sustainable synthesis involving chemical transformations that are very difficult or practically impossible to conduct using conventional batch conditions. With the aim to show how flow microreactor technology and flash chemistry could contribute to the development of a sustainable organic synthesis, very recent examples have been selected and will be discussed here. In the context of green chemistry [11], protecting-group free organic synthesis has received particular attention in the last years, because of atom economy [12–15] and reduction of synthetic steps [16]. It has been demonstrated by Yoshida that protecting-group-free synthesis could be feasible using flash chemistry and microreactor technology [17–18]. Recently, Yoshida and co-workers developed flash methods for the generation of highly unstable carbamoyl anions, such as carbamoyllithium, using a flow microreactor system [19]. In particular, they reported that starting from different substituted carbamoyl chloride **1** and lithium naphthalenide (LiNp) it was possible to generate the corresponding carbamoyllithium **2**, that upon trapping with different electrophiles provided several amides and ketoamide **3** (Scheme 1).

**Scheme 2:**
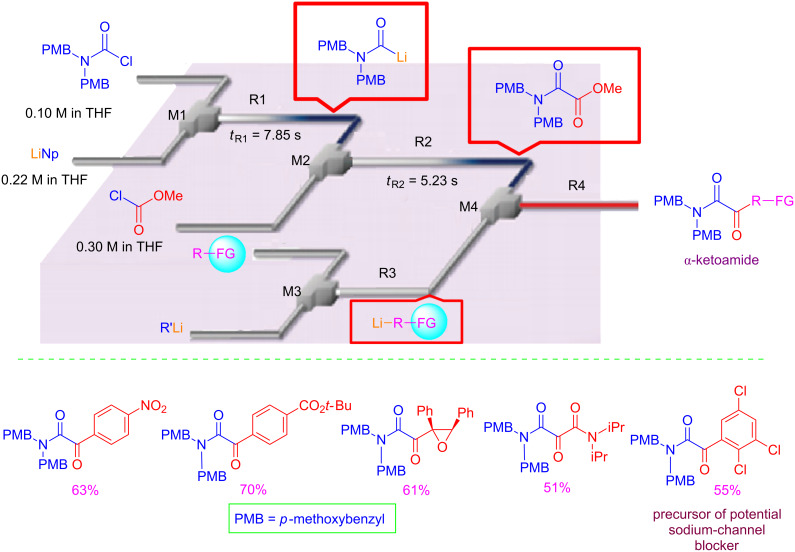
Flow synthesis of functionalized α-ketoamides.

The use of an integrated microflow system allowed the preparation of functionalized α-ketoamides by a three-component reaction between carbamoyllithium, methyl chloroformate and organolithium compounds bearing sensitive functional groups (i.e., NO_2_, COOR, epoxide, carbonyl) (Scheme 2).

It should be stressed that this kind of sequential transformations are practically impossible to perform using conventional batch chemistry because of the incompatibility of sensitive functional groups with organolithiums, and because of the high chemical and thermal instability of the intermediates.

**Scheme 3:**
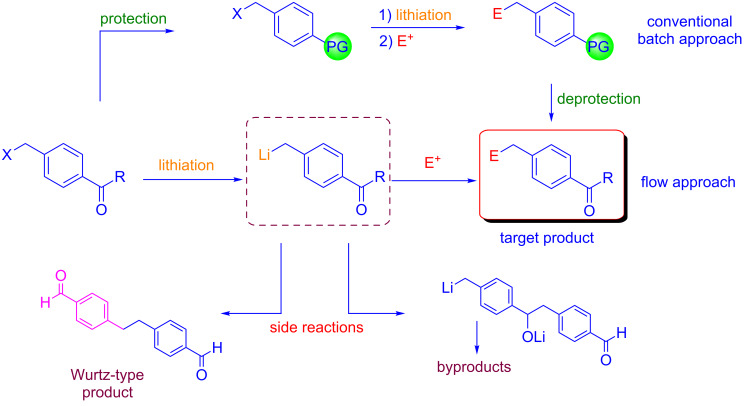
Reactions of benzyllithiums.

In 2015 Yoshida reported another remarkable finding on the use of protecting-group-free organolithium chemistry. In particular, the flash chemistry approach was exploited for generating benzyllithiums bearing aldehyde or ketone carbonyl groups [20]. This reaction could be problematic for two reasons: a) the competing Wurtz-type coupling, (i.e., the coupling of benzyllithiums with the starting benzyl halides); b) the nucleophilic attack of organolithium species to aldehyde or ketone carbonyl groups (Scheme 3).

**Scheme 4:**
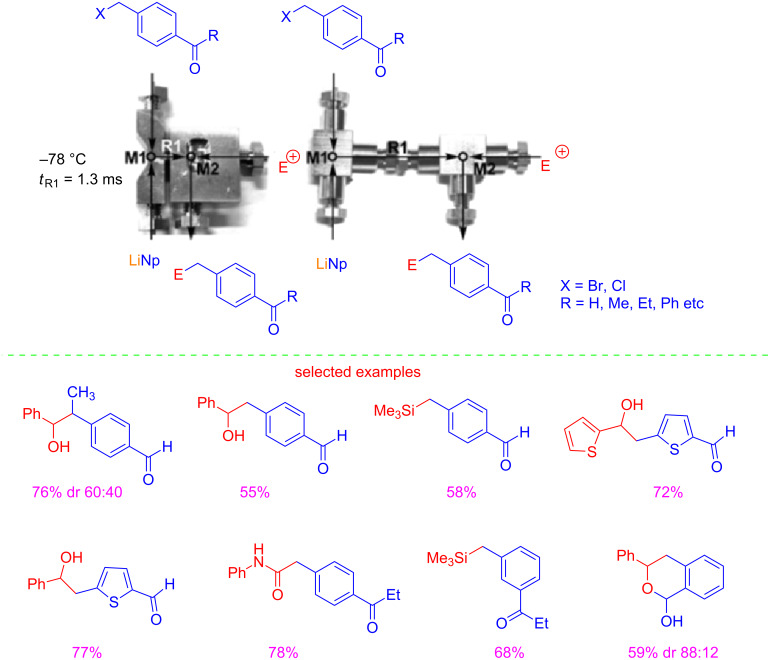
Trapping of benzyllithiums bearing carbonyl groups enabled by a flow microreactor. (Adapted with permission from [18], copyright 2015 The Royal Society of Chemistry).

The authors reported that the extremely fast micromixing avoided undesired Wurtz-type coupling [21–22]. It is well known, that competitive reactions can be controlled or even avoided under fast micromixing [23–27]. Moreover, high-resolution residence time control was essential for survival of carbonyl groups. In fact, this transformation can be achieved only with a residence time of 1.3 ms at −78 °C. Under these flow conditions, the aldehyde or ketone carbonyl moiety can survive the nucleophilic organolithium attack. Remarkably, the flow microreactor system allowed also the generation of benzyllithiums at 20 °C, rather than under cryogenic (−95 °C) conditions adopted with a conventional batch protocol. In addition, THF could be used in place of mixed solvents (Et_2_O/THF/light petroleum). Under the optimized conditions, the reactions of benzyllithiums with different electrophiles, gave adduct products in good yields (Scheme 4).

Another useful aspect of the flash chemistry relies on the possibility to generate highly reactive intermediates, such as halomethyllithium carbenoids, that need to be used under internal-quenching technique in batch mode. In 2014, the first example of effective external trapping of a reactive chloromethyllithium (CML) has been reported [28]. α-Haloalkyllithiums are a useful class of organometallic reagents widely employed in synthetic chemistry. In fact, they allow the direct homologation of carbonyl compounds and imines leading to β-halo-alcohols and amines that are useful building blocks [29–31]. This work represents a remarkable example of flash chemistry, and has elements of sustainability considering that in batch macroreactors, in order to avoid metal-assisted α-elimination, in situ quenching, an excess of reagents, and very low temperature are required [32–33].

**Scheme 5:**
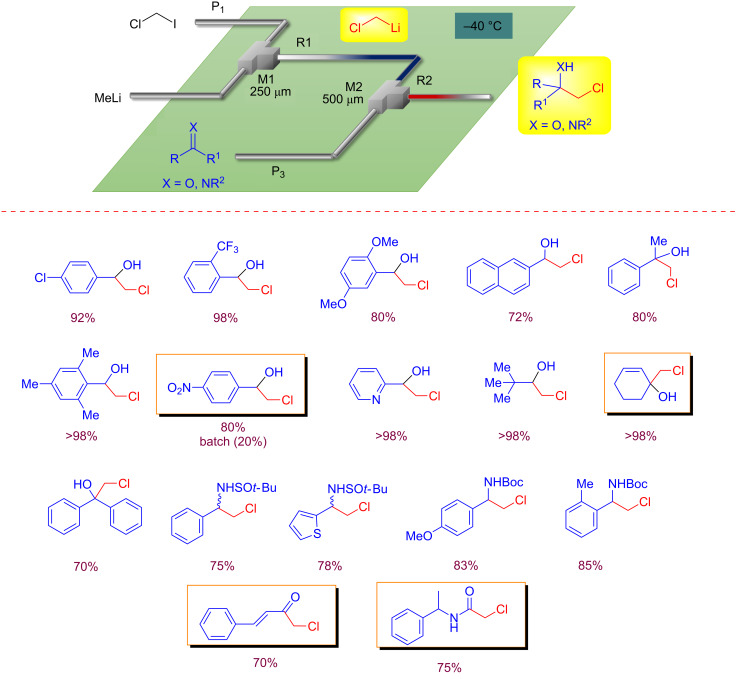
External trapping of chloromethyllithium in a flow microreactor system.

Running the reaction in a flow system at −40 °C, by using residence times between 0.18–0.31 s high yields of homologated products have been obtained under external quenching conditions (Scheme 5).

**Scheme 6:**
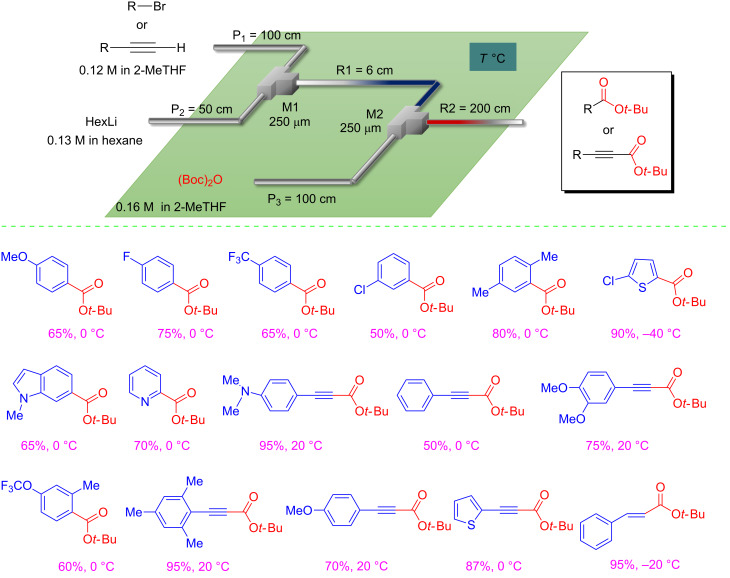
Scope for the direct *tert*-butoxycarbonylation using a flow microreactor system.

The results described above nicely show the potential, as green technology, of flow microreactor systems for synthetic processes involving highly unstable intermediates. Another nice example on the use of microreactor technology for the development of sustainable chemical processes, is represented by the direct introduction of the *tert*-butoxycarbonyl group into organometallic reagents [34]. The reaction between organolithium reagents and di-*tert*-butyl dicarbonate run under flow conditions, allowed a straightforward preparation of several *tert*-butyl esters. The use of a flow process resulted more efficient, versatile and sustainable compared to batch. Moreover, this operationally simple procedure complements well with the already available strategies for the preparation of *tert*-butyl esters, avoiding the use of inflammable and explosive gaseous isobutylene [35], the use of harsh conditions [36], the use of peroxides [37], the use of toxic gas such as CO or transition metals [38–42]. The flow process, for the direct C-*tert*-butoxycarbonylation of organolithiums, has been optimized in a green solvent such as 2-MeTHF by a precise control of the residence time, and without using cryogenic conditions (Scheme 6). In addition, many organolithiums were generated from the corresponding halo compounds by a halogen/lithium exchange reaction using hexyllithium as a more sustainable base [43–44].

The concept of flash chemistry has been successfully employed for outpacing fast isomerization reactions. The accurate control of the residence time, realized in a microreactor, could suppress or avoid isomerization of unstable intermediates. This is often unavoidable when the same reactions are run in batch mode [45–47].

**Scheme 7:**
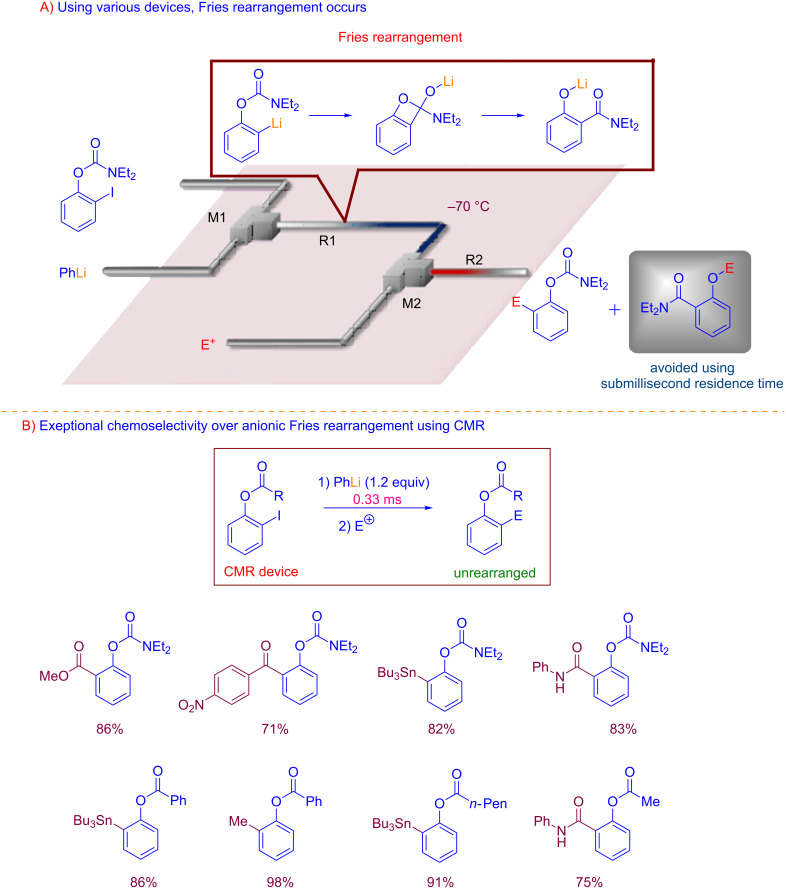
Control of anionic Fries rearrangement reactions by using submillisecond residence time. (Adapted with permission [43], copyright 2016 American Association for the Advancement of Science).

Yoshida and Kim recently provided an astonishing example on the potential of flash chemistry in controlling fast isomerization of organolithiums [48]. The authors designed a chip microreactor (CMR), able to deliver a reaction time in the range of submilliseconds (0.33 ms) under cryogenic conditions. By using such an incredible short residence time, it was possible to overtake the very rapid anionic Fries rearrangement, and chemoselectively functionalize *ortho*-lithiated aryl carbamates (Scheme 7).

**Figure 2:**
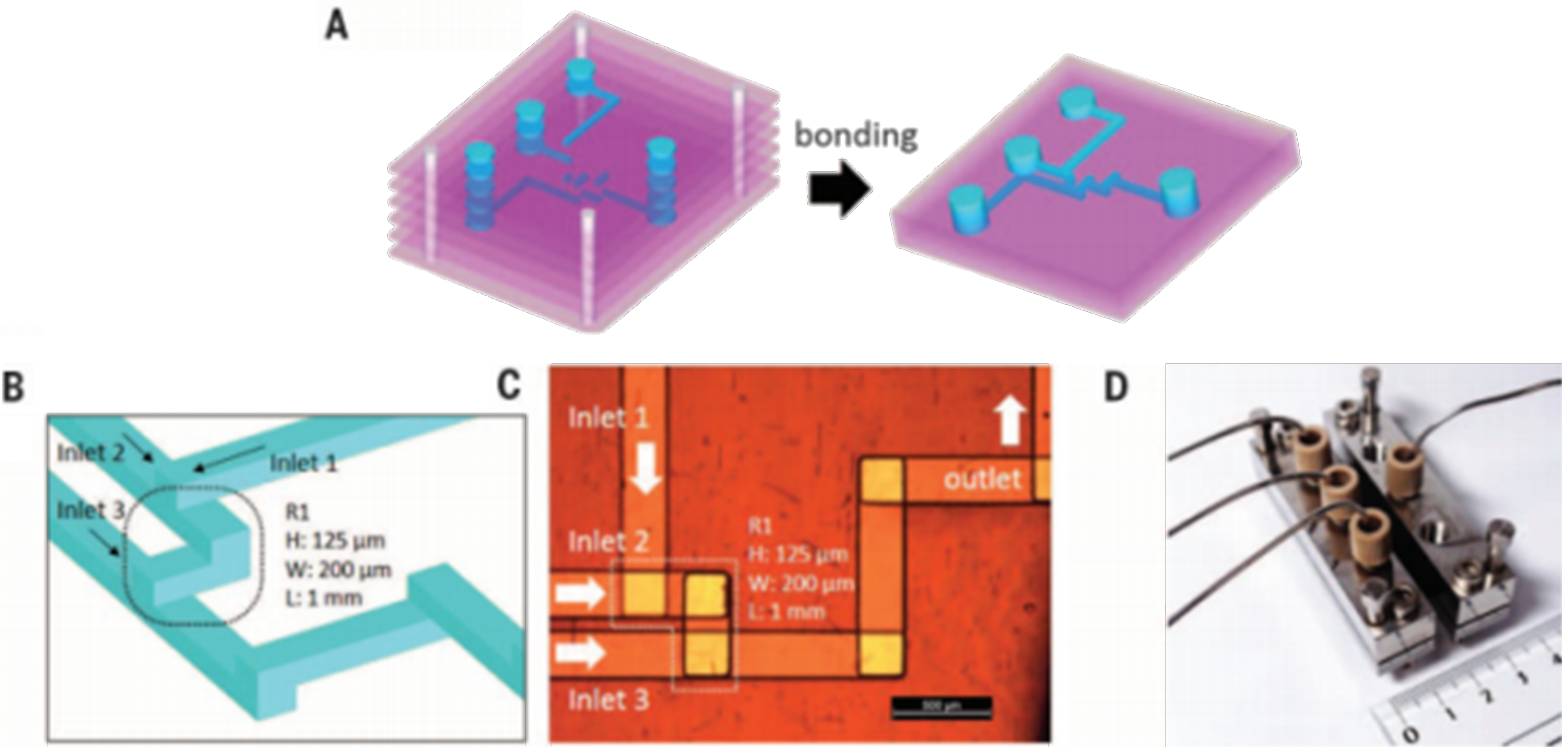
Chip microreactor (CMR) fabricated with six layers of polyimide films. (Reproduced with permission from [43], copyright 2016 American Association for the Advancement of Science).

This CMR has been developed choosing a fluoroethylene propylene–polymide film hybrid for fabrication because this material offers exceptional physical toughness at low temperature and high pressure as well as chemical inertness. The most relevant aspect of this microreactor, concerns the 3D design of the mixing zone (Figure 2). The mixing efficiency was evaluated on the basis of computational fluids dynamics (CFD). The simulation results showed that serpentine 3D-structured channels (Figure 2), possessing five turns after each mixing point in a total length of 1 mm, was able to deliver the highest mixing efficiency. The inner volume for the reactor was of 25 μL. This CMR provides mixing efficiency levels of 95% with a total flow rates of 7.5 mL/min corresponding to a residence time of about 0.3 milliseconds.

To show the potential use of this microdevice in organic synthesis, the synthesis of Afesal [49], a biologically active compound having anthelmintic activity was reported as application.

This outstanding result by Yoshida and Kim, demonstrates how microdevices and flash chemistry could contribute to the development of new sustainable synthetic strategies, and how microreactor technology could help in taming the reactivity of unstable species [50].

### Contribution of continuous-flow metal-, organo-, and photocatalysis in green chemistry

The development of continuous-flow catalysis is appealing because it combines the advantages of a catalytic reaction with the benefits of flow microreactors. Under homogeneous conditions a soluble catalyst, which flows through the reactor together with the reactants, is employed. At the end of the process, a separation step would be required in order to remove the catalyst and byproducts. On the other hand, heterogeneous catalysis is widely used in the synthesis of bulk and fine chemicals. In a continuous-flow process, the catalyst can be fixed on a suitable hardware, and the reaction mixture allowed to flow through the system. The use of recyclable catalysts in continuous-flow conditions represents an innovative strategy for the development of more environmentally friendly synthesis. In the last decade, organic photochemistry got a sort of renaissance, emerging as useful approach in modern sustainable and green synthesis.

**Scheme 8:**
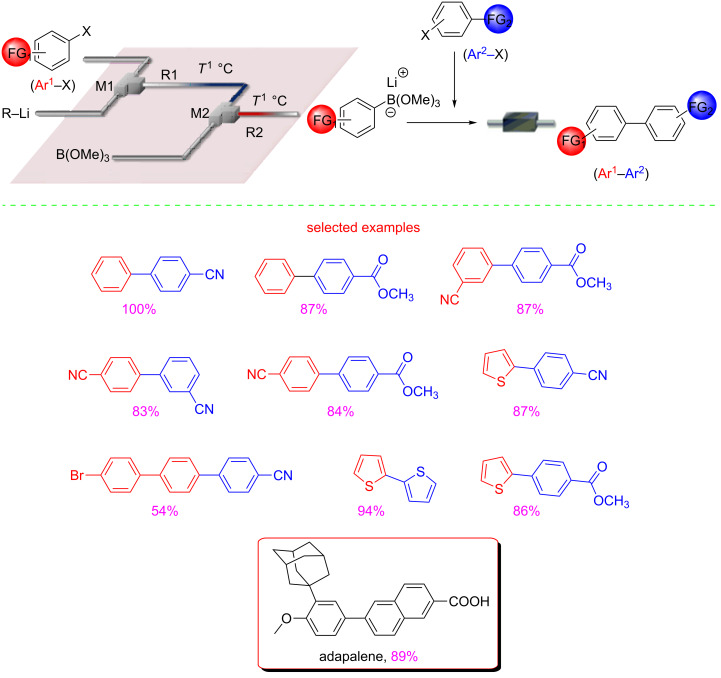
Flow microreactor system for lithiation, borylation, Suzuki–Miyaura coupling and selected examples of products.

Concerning the heterogeneous catalysis with palladium, practical procedures for recovering and reusing of the catalysts have been recently reported [51–53]. A versatile Pd-catalysed synthesis of polyfunctionalized biaryls, using a flow microreactor, has been recently reported by Yoshida [54]. Using the integrated microflow system reported in Scheme 8, arylboronic esters were prepared by a lithiation/borylation sequence, and used in a Suzuki–Miyaura coupling in a monolithic reactor. A remarkable aspect of the process was the use of an integrated supported monolithic Pd(0) catalyst that allowed to perform cross-coupling reactions in continuous flow mode (Scheme 8).

This integrated microflow system allow to handle the borylation of aryl halides (Ar^1^X), and the subsequent Suzuki–Miyaura coupling using different aryl halide (Ar^2^X). Without requiring the protection of sensitive functionalities, running the flow system using a residence time (*t*_R_) of about 4.7 min at a temperature above 100 °C, high yields of coupling products were obtained. Noteworthy, the Suzuki–Miyaura coupling did not require the use of a base. The authors applied the presented method to the synthesis of adapalene, used in the treatment of acne, psoriasis, and photoaging.

**Scheme 9:**
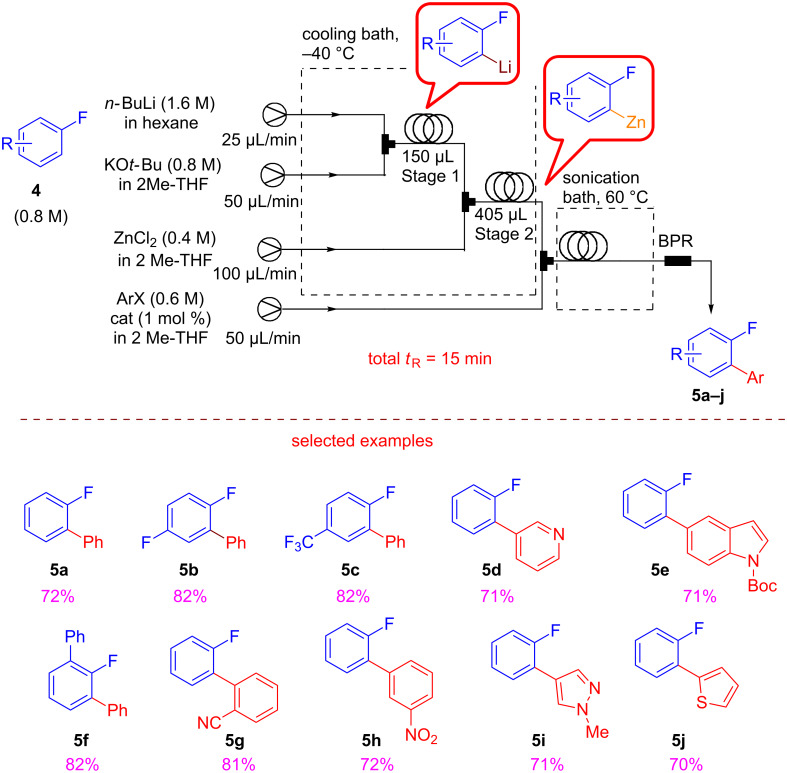
Experimental setup for the flow synthesis of 2-fluorobi(hetero)aryls by directed lithiation, zincation, and Negishi cross-coupling. (Adapted with permission from [53], copyright 2016 Wiley-VCH Verlag GmbH & Co. KGaA, Weinheim).

Fluorinated aromatic compounds are extremely important in agrochemical, pharmaceutical and medicinal fields [55–58]. Buchwald and co-workers suggested a telescoped homocatalysis procedure consisting of a three-step sequence (metalation, zincation and Negishi cross-coupling) which furnishes an easy access to a variety of functionalized 2-fluorobiaryl and heteroaryl products (Scheme 9) [59]. This strategy is rightfully considered green because it guarantees the employment of readily available and cheap starting materials, the safe handling of highly thermally unstable or dangerous intermediates, and the use of higher temperature with respect to the batch mode in which the proposed reactions have to be carried out at −78 °C.

The use of 2-MeTHF as greener solvent, contributes to further validate the green procedure. The 2-MeTHF solutions of fluoroarenes **4** together with the hexane solution of *n*-BuLi were pumped into the flow system at −40 °C. The generated organozinc intermediate meets the solution of haloarenes and the catalyst, leading to the formation of the desired products **5a–j** (Scheme 9). Noteworthy, the homogeneous catalysis requires only 1% of the XPhos-based palladium catalyst. A sonication bath was employed to prevent clogging and the reaction required a residence time of 15 min.

**Scheme 10:**
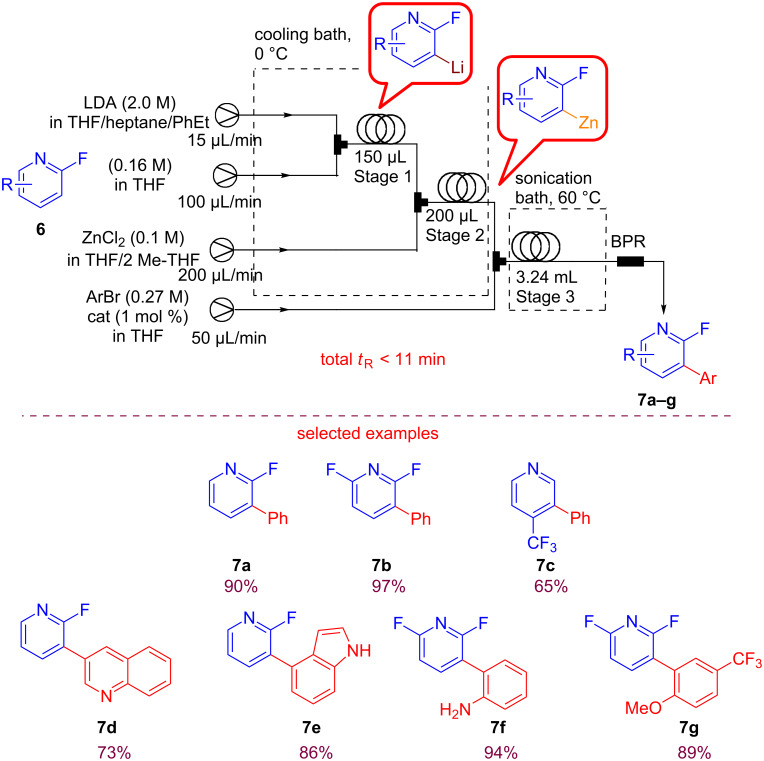
Experimental setup for the coupling of fluoro-substituted pyridines. (Adapted with permission from [53], copyright 2016 Wiley-VCH Verlag GmbH & Co. KGaA, Weinheim).

Next, they turned their attention to the arylation of fluoro-substituted pyridines. The regioselective lithiation of halopyridines with lithium diisopropylamide (LDA) was conducted under mild conditions on substrate **6** (Scheme 10). The addition of a little amount of THF was necessary in order to avoid clogging and the tendency of the lithiated intermediate to eliminate.

**Scheme 11:**
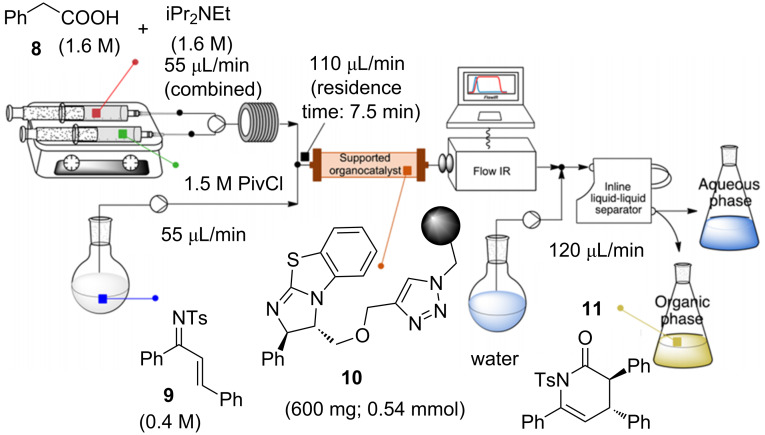
Continuous flow process setup for the preparation of **11** (Reproduced with permission from [54], copyright 2015 American Chemical Society).

The optimized conditions were suitable for the functionalization of 2-fluoropyridine, 2,6- difluoropyridine and 4-(trifluoromethyl)pyridine leading to products **7a–g** reported in Scheme 10. Another promising field is the sustainable flow organocatalysis, and recently Pericàs reported an interesting synthesis and application of a recyclable immobilized analogue of benzotetramisole (BMT) used in a catalytic enantioselective Michael addition/cyclization reactions under continuous-flow conditions (Scheme 11) [60].

Resin-bound catalyst **10** was swollen with dichloromethane in a medium-pressure chromatography column used as a reactor. Dichloromethane solutions of substrate **9** reacted with the mixed phenylacetic pivalic anhydride (deriving from phenylacetic acetic (**8**) and pivaloyl chloride) inside the catalytic reactor producing the expected products **11**. This ingenious system was equipped with an in-line FTIR probe, for monitoring the transformation, and an in line liquid–liquid separator to avoid tedious work-up procedures, thus saving solvents, resources and optimizing work times. This system was demonstrated to work for 11 h with higher conversion and enantioselectivity (er >99.9%) in comparison to the batch mode [61]. Pericàs and co-workers taking advantage of the high catalytic activity, robustness and recyclability of the supported catalyst, performed also straightforward gram synthesis of target compounds.

**Scheme 12:**
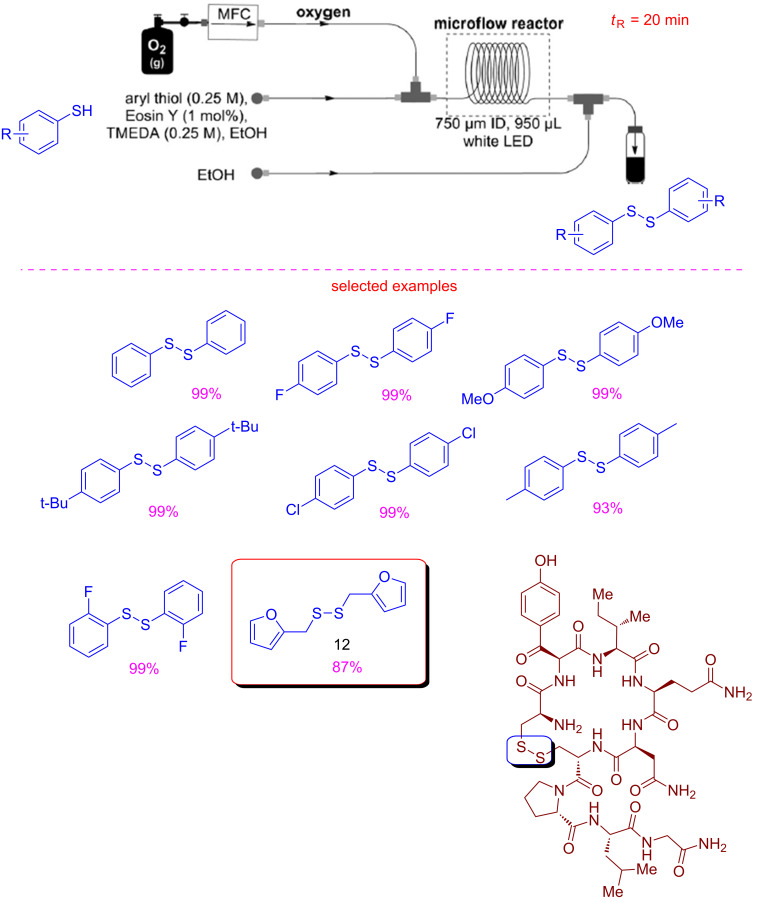
Continuous-flow photocatalytic oxidation of thiols to disulfides.

In the context of photocatalysis and oxidations using flow microreactors [62–63], Noël reported a metal-free photocatalytic aerobic oxidation of thiols to disulfides under continuous-flow conditions [64]. Disulfides are useful molecules employed as drugs, anti-oxidants or pesticides as well as rubber vulcanizating agents [65]. Symmetric disulfides are generally obtained by oxidative coupling of thiols [66]. Noël and co-workers set up a microflow system equipped with a mass flow controller (MFC) able to introduce pure oxygen as the oxidant to oxidize a solution of thiol containing 1% of Eosin Y. The flow stream was exposed to white LED light in order to activate the reaction, and a dilution with pure EtOH was needed at the output to avoid clogging (Scheme 12). The residence time of 20 min guaranteed a limited irradiation time and high purity of the products.

The disulfides were obtained with excellent yields, and the process was executed on challenging thiols as in the case of disulfide **12** (Scheme 12), used as food flavour additive [67]. To demonstrate the usefulness of the flow methodology, and its applicability, the photocatalytic aerobic oxidation of a peptide to obtain oxytocin in continuous flow was reported (Scheme 12). Full conversion was achieved in water with 200 s of residence time.

**Scheme 13:**
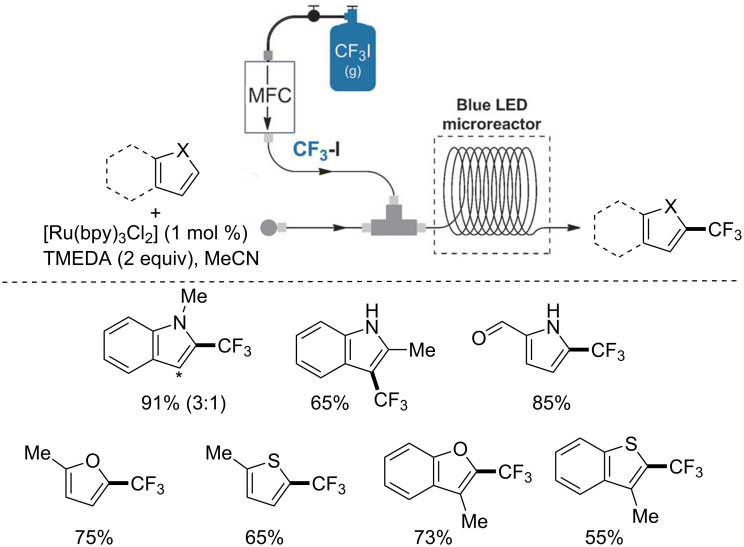
Trifluoromethylation by continuous-flow photoredox catalysis.

Noël optimized, for the first time, a trifluoromethylation of aromatic heterocycles by continuous-flow photoredox catalysis. The process benefited from the use of microreactor technology and readily available photocatalysts. The process was also employable for perfluoroalkylation. The developed process occurred in less time with respect to batch mode, and under milder conditions. The set-up of the reactor allowed for the use of gaseous CF_3_I by means of a mass flow controller. Selected examples of trifluoroalkylated products are reported in Scheme 13 [68].

**Scheme 14:**
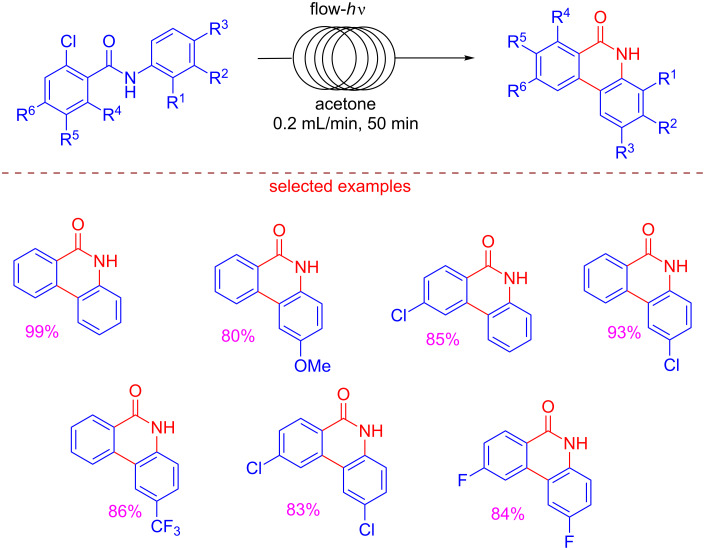
Flow photochemical synthesis of 6(5*H*)-phenanthridiones from 2-chlorobenzamides.

Tranmer reported a “traceless reagents” chemistry with the continuous-flow photosynthesis of 6(5*H*)-phenanthridinones, poly(ADP-ribose) polymerase (PARP) inhibitors [69]. The relevance of the work resides in the use of green solvents, the absence of heavy metals, the use of convenient temperatures, and the increased safety by eliminating UV-exposure locating the UV lamp within the microreactor. Hazard of fires caused by the hot UV lamps approaching the auto-ignition temperature of flammable solvents, very often underestimated, is totally prevented thanks to a specific cooling system. 2-Halo-*N*-arylbenzamides were converted into 6(5*H*)-phenanthridinones by a photocyclization reaction. In order to run this step, a flow system with a photochemical reactor equipped with a medium pressure Hg lamp and 10 mL reactor coil, was employed. Good yields were obtained from different 2-chlorobenzamides disclosing that either electron-donating or electron withdrawing *ortho*-substituents were tolerated (Scheme 14).

**Scheme 15:**
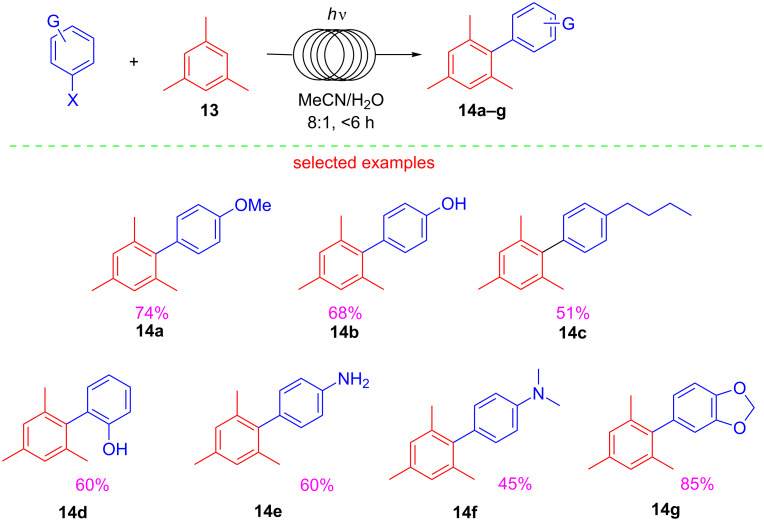
Synthesis of biaryls **14a–g** under photochemical flow conditions.

A metal- and catalyst-free arylation procedure carried out under continuous-flow conditions was recently reported by Fagnoni [70]. This photochemical process allowed for the preparation of a wide range of synthetic targets by Ar–Csp^3^, Ar–Csp^2^ and Ar–Csp bond-forming reactions. The use of a photochemical flow reactor, consisting of a polyfluorinated tube reactor wrapped around a 500 W Hg lamp, allowed to overcome batch limitations paving the way for metal-free arylation reactions via phenyl cations. Derivatives **14a–g** were prepared with this greener flow approach (Scheme 15) starting from mesitylene **13**, and haloarenes using short irradiation times (<6 h), and a 5:1 MeCN/H_2_O mixture.

The reported results show how photochemistry hold the potential to become a green tool for the development of sustainable photochemical flow synthesis.

### Hazardous chemistry by using green and sustainable continuous-flow microreactors

We have already shown how continuous-flow technology could play an important role in improving chemical processes [5,71], providing different advantages over traditional batch mode. However, the hazardous nature of some chemicals makes handling at conventional lab or industrial scale difficult. The use of microreactors and continuous-flow chemistry offers the possibility to perform reactions using dangerous or hazardous materials that cannot be used in batch mode. In other word, syntheses previously "forbidden" for safety reasons, such as those involving diazo compounds, hydrazine, azides, phosgene, cyanides and other hazardous chemicals could be performed with relatively low risk using flow technology [72–76].

Several research groups investigated this aspect, as highlighted by several available reviews [77–78]. Here we describe very recent reports with the aim to highlight the potential of flow chemistry in the field of hazardous chemistry under a greener perspective.

**Scheme 16:**
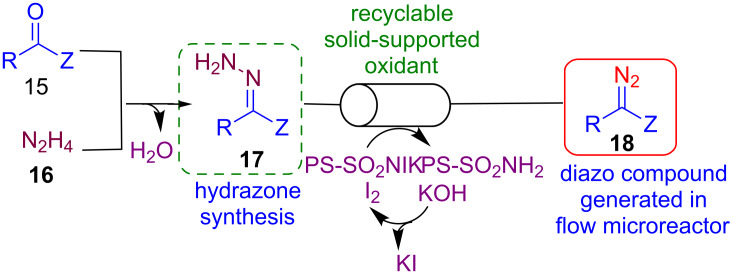
Flow oxidation of hydrazones to diazo compounds.

Diazo compounds are recognized as versatile reagents in organic synthesis. Nevertheless, diazo compounds are also considered highly energetic reagents [79–80]. For this reason, the in situ generation of such reagents has been investigated under flow conditions. Moody and co-workers reported a new method for the in situ generation of diazo compounds as precursors of highly reactive metal carbenes (Scheme 16) [81].

As reported in Scheme 16, diazo species **18** could be generated from simple carbonyls **15** and hydrazine (**16**). Intermediate hydrazones **17** can be converted into the corresponding diazo compounds by oxidation using a recyclable oxidant based on *N*-iodo-*p*-toluenesulfonamide potassium salt. The possibility to regenerate a functionalized resin by simple washing with aqueous KI_3_/KOH solution makes the process more sustainable. This method produces KI solution as waste, and it is an alternative way for the direct oxidation of hydrazones, that often requires the use of heavy metals such as HgO, Pb(OAc)_4_ and AgO [82–83].

**Scheme 17:**
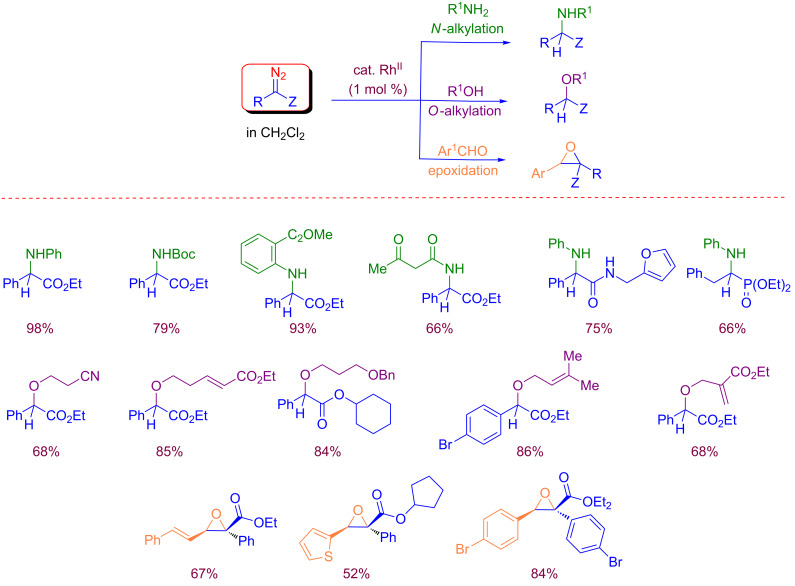
Synthetic use of flow-generated diazo compounds.

The diazo compounds could be collected as solution in dichloromethane at the output of the flow system, and obtained sufficiently pure for further use without requiring handling or isolation. Further mixing of solutions containing diazo derivatives to a solution containing a Rh(II) catalyst, and reactants such as amines, alcohols or aldehydes led to a wide range of products as reported in Scheme 17.

**Scheme 18:**
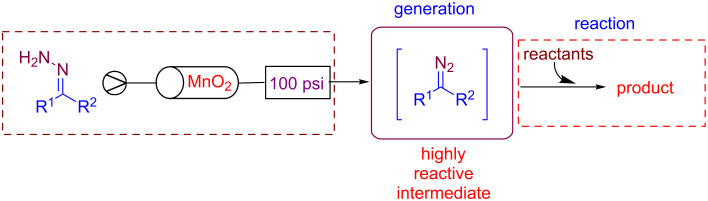
Ley’s flow approach for the generation of diazo compounds.

Ley's group developed several continuous-flow approaches for generating diazo species from hydrazones [84–85]. Under flow conditions, diazo compounds were reacted with boronic acids in order to generate reactive allylic and benzylic boronic acids further employed for iterative C–C bond forming reactions [86]. The generation of unstable diazo species was possible using a cheap, recyclable and less toxic oxidant, MnO_2_. The flow stream was accurately monitored by in-line FTIR spectroscopy in order to maximize the formation of the diazo compound (Scheme 18) [87].

**Scheme 19:**
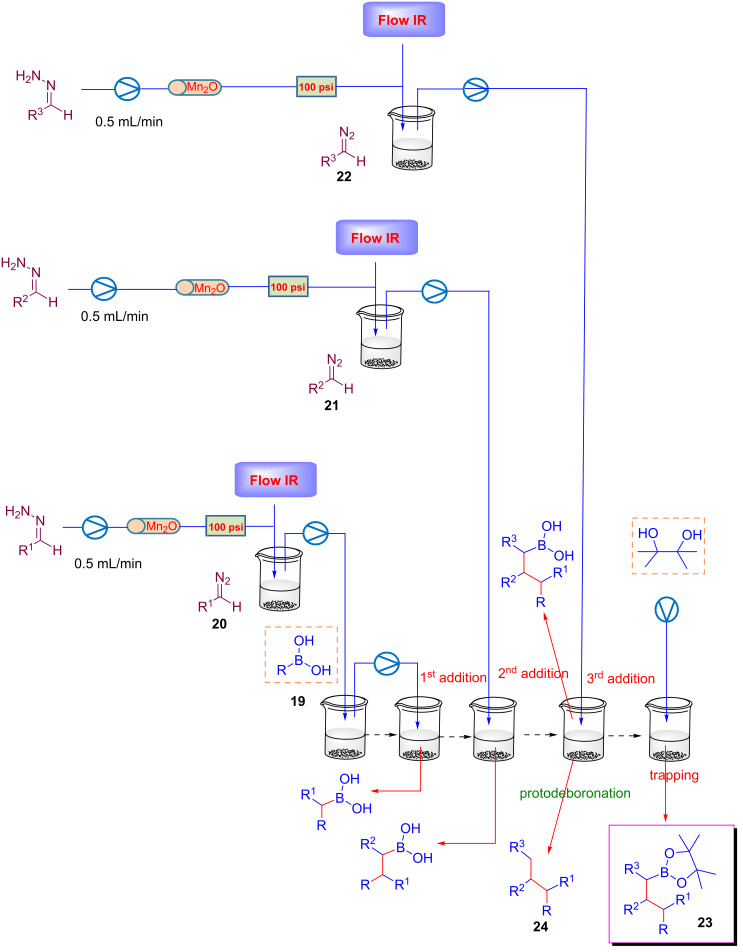
Iterative strategy for the sequential coupling of diazo compounds.

Starting from this initial investigation, Ley and co-workers developed an elegant application of this strategy for a sequential formation of up to three C–C bonds in sequence, by an iterative trapping of boronic acid species. The sequence starts with the reaction of diazo compound **20**, generated under flow conditions, and boronic acid **19** (Scheme 19). Further sequential coupling with diazo compounds **21** and **22** led to boronates **23** or protodeboronated products **24** at the end of the sequence (Scheme 19).

**Scheme 20:**
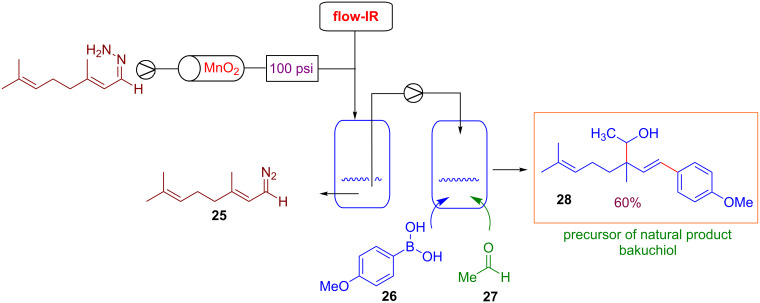
Integrated synthesis of Bakuchiol precursor via flow-generated diazo compounds.

With the aim to exploit the versatility of this approach, Ley and co-workers reported the allylations of carbonyl electrophiles such as aldehydes using the above reported strategy for the generation of allylboronic acids. The flow protocol considers the reaction of diazo compounds **25** (generated in flow) with boronic acid **26** and aldehyde **27** (Scheme 20). By this new iterative coupling it was possible to obtain alcohols as products. The usefulness of the method was demonstrated with the preparation in good yield (60%) of a precursor of the natural product bakuchiol **28** (Scheme 20) [88].

**Scheme 21:**
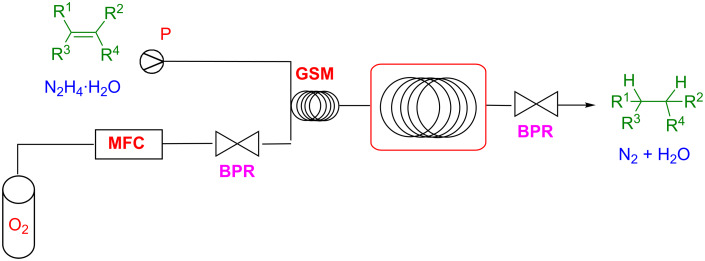
Kappe’s continuous-flow reduction of olefines with diimide.

The microreactor technology offers the advantage to handle hazardous components such as hydrazine and molecular oxygen, which represent alternative reagents for selective reduction of C=C double bonds. In fact, combination of hydrazine hydrate (N_2_H_4_·H_2_O) and O_2_ provide diimide (HN=NH) as reducing agent. Nevertheless, this strategy is rarely used in traditional batch chemistry for safety reason. Kappe and co-workers recently developed a reduction of the alkene to the corresponding alkane, by a catalyst-free generation of diimide by oxidation of hydrazine monohydrate (N_2_H_4_·H_2_O) with molecular oxygen [89–90]. The flow system set-up is reported in Scheme 21*,* and consists in a HPLC pump for delivering the alkene and hydrazine monohydrate, while O_2_ was delivered by a mass-flow controller (MFC) from a standard compressed-gas cylinder. After combination of the reagent streams, the resulting segmented flow was pumped through a heated residence unit (RTU) consisting in a fluorinated tube with low gas permeability (Scheme 21).

**Scheme 22:**
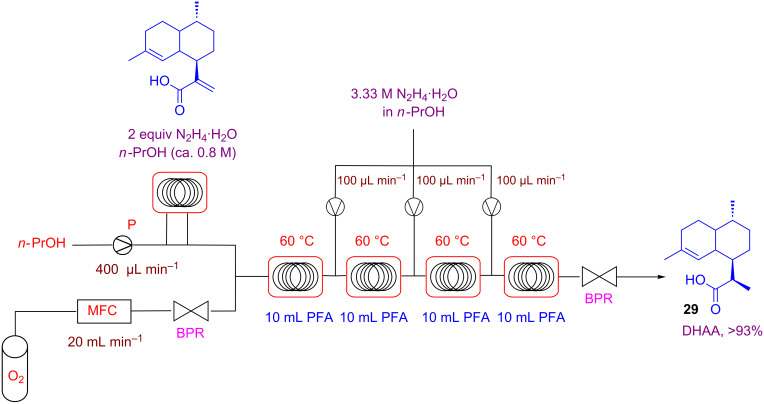
Multi-injection setup for the reduction of artemisinic acid.

The flow system reported in Scheme 21 was able to reduce alkenes with high yields and selectivity by using residence times in the range of 10 to 30 min at 100 °C, and by employing a slight excess of hydrazine. Importantly, this strategy is compatible with sensitive functional groups such as silyl ether, halogenes, and benzyl groups. A very nice application of this approach was the highly selective reduction of artemisinic acid to dihydroartemisinic acid, which are of interest in the synthesis of the antimalarial drug artemisinin. This industrially relevant reduction was executed by using O_2_ at 20 bar, four residence units at 60 °C and consecutive feedings with N_2_H_4_·H_2_O in order to obtain full conversion in dihydroartemisinic acid (**29**, DHAA, Scheme 22).

### Continuous-flow sustainable production of APIs

**Scheme 23:**
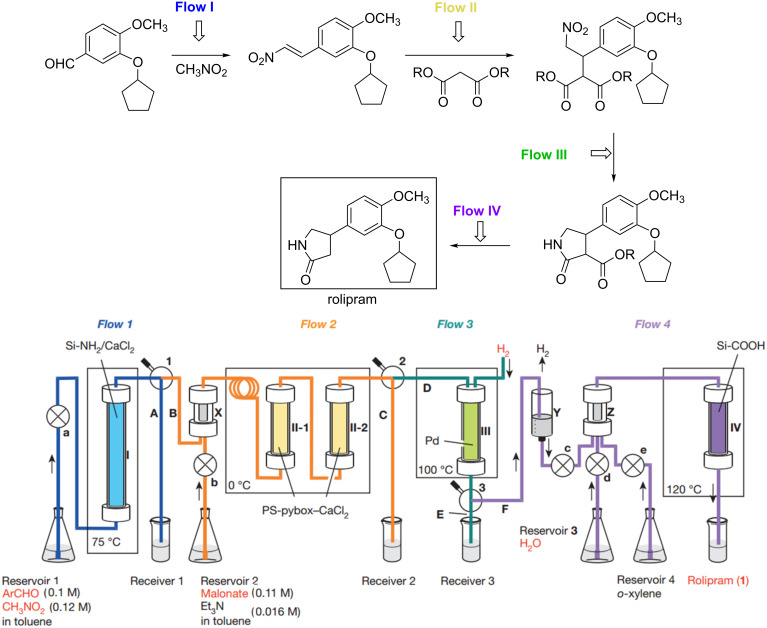
Flow reactor system for multistep synthesis of (*S*)-rolipram. Pumps are labelled a, b, c, d and e; Labels A, B, C, D, E and F are flow lines. X are molecular sieves; Y is Amberlyst 15Dry; Z is Celite. (Reproduced with permission from [84], copyright 2015 Nature Publishing Group).

With the aim to demonstrate the potential of microreactor technology and flow chemistry in sustainable synthesis, recent outstanding “proof of concepts” will be described. Kobayashi and co-workers reported a multistep continuous-flow synthesis of a drug target via heterogeneous catalysis. The developed process not requiring any isolation of intermediates, separation of the catalyst or other work-up procedures can be considered sustainable [91]. The syntheses of (*S*)-rolipram and a γ-aminobutyric acid (GABA) derivative were accomplished. Readily available starting materials and columns containing chiral heterogeneous catalysts to produce enantioenriched materials were employed. It is worth mentioning that this work represents a very nice example on the use of chiral catalysis in a multistep flow synthesis of a drug target on gram scale. The multistep synthesis of (*S*)-rolipram reported in Scheme 23 begins from a benzaldehyde derivative which undergoes a Henry-type reaction with nitromethane in the first flow step (Flow I). The resulting nitroalkene undergoes an asymmetric addition catalyzed by a supported PS–(*S*)-pybox–calcium chloride catalyst at 0 °C using two columns (Flow II). This is the enantio-determining step of the process. The stereochemistry of the adduct can be simply switched to the opposite enantiomer, by using the enantiomeric supported catalyst PS–(*R*)-pybox–calcium chloride. The enantiomeric excess of the products was about 96%. Two more steps consisting in a Pd-catalyzed hydrogenation reaction and a decarboxylation (Flow III and Flow IV) led to the target (*S*)-rolipram in 50% overall yield. The systems was designed in order to keep the level of the palladium in solution as low as possible (<0.01 ppm).

**Figure 3:**
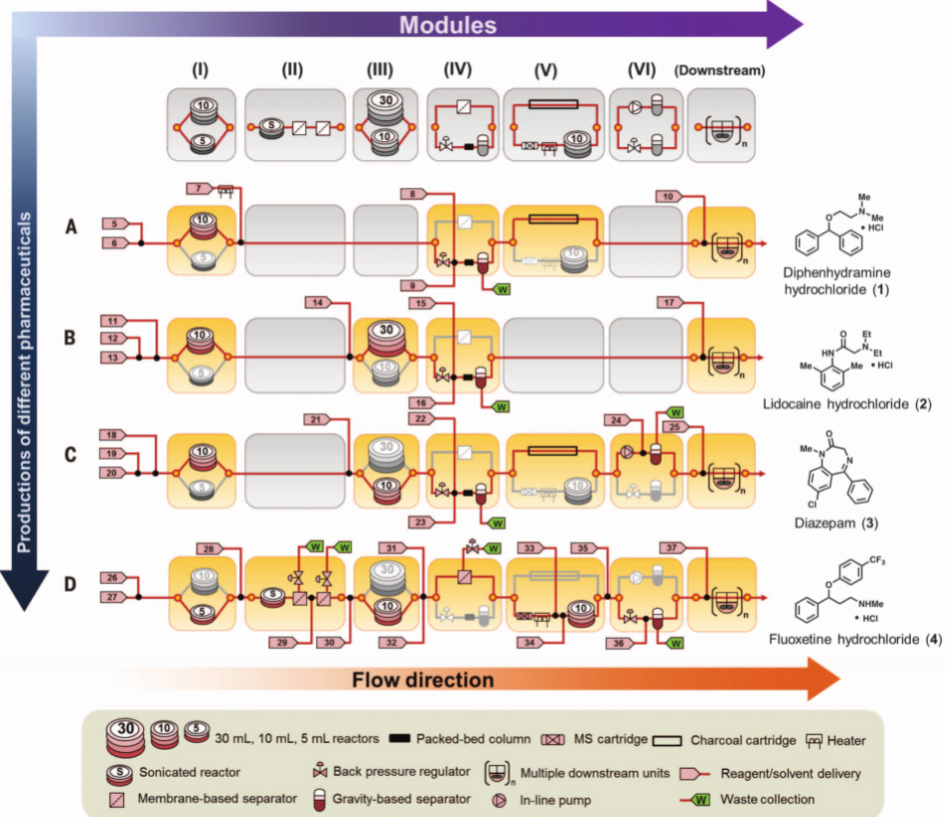
Reconfigurable modules and flowcharts for API synthesis. (Reproduced with permission from [85], copyright 2016 American Association for the Advancement of Science).

Another outstanding proof of concept, which demonstrates the potential of flow chemistry for sustainable pharmaceutical manufacturing, has been recently reported by Jensen and his research team. The research team set up a compact and reconfigurable manufacturing platform for the continuous-flow synthesis and formulation of active pharmaceutical ingredients (APIs) [92]. The “mini” plant (reported in Figure 3) was very compact in size [1.0 m × 0.7 m × 1.8 m, (W × L × H)], and low-weighing (about 100 kg) and was able to perform complex multistep synthesis, work-up procedures as well as purification operations such as crystallization. This platform was also equipped with devices for real-time monitoring and final formulation of high purity APIs. For the preparation of target molecules, commercially available starting materials were employed. The platform was tested for the production and supply of hundreds to thousands doses per day of diphenhydramine hydrochloride, lidocaine hydrochloride, diazepam and fluoxetine hydrochloride.

Remarkably, for future applications of the platform, the produced medicines also met the U.S. Pharmacopeia standards.

**Figure 4:**
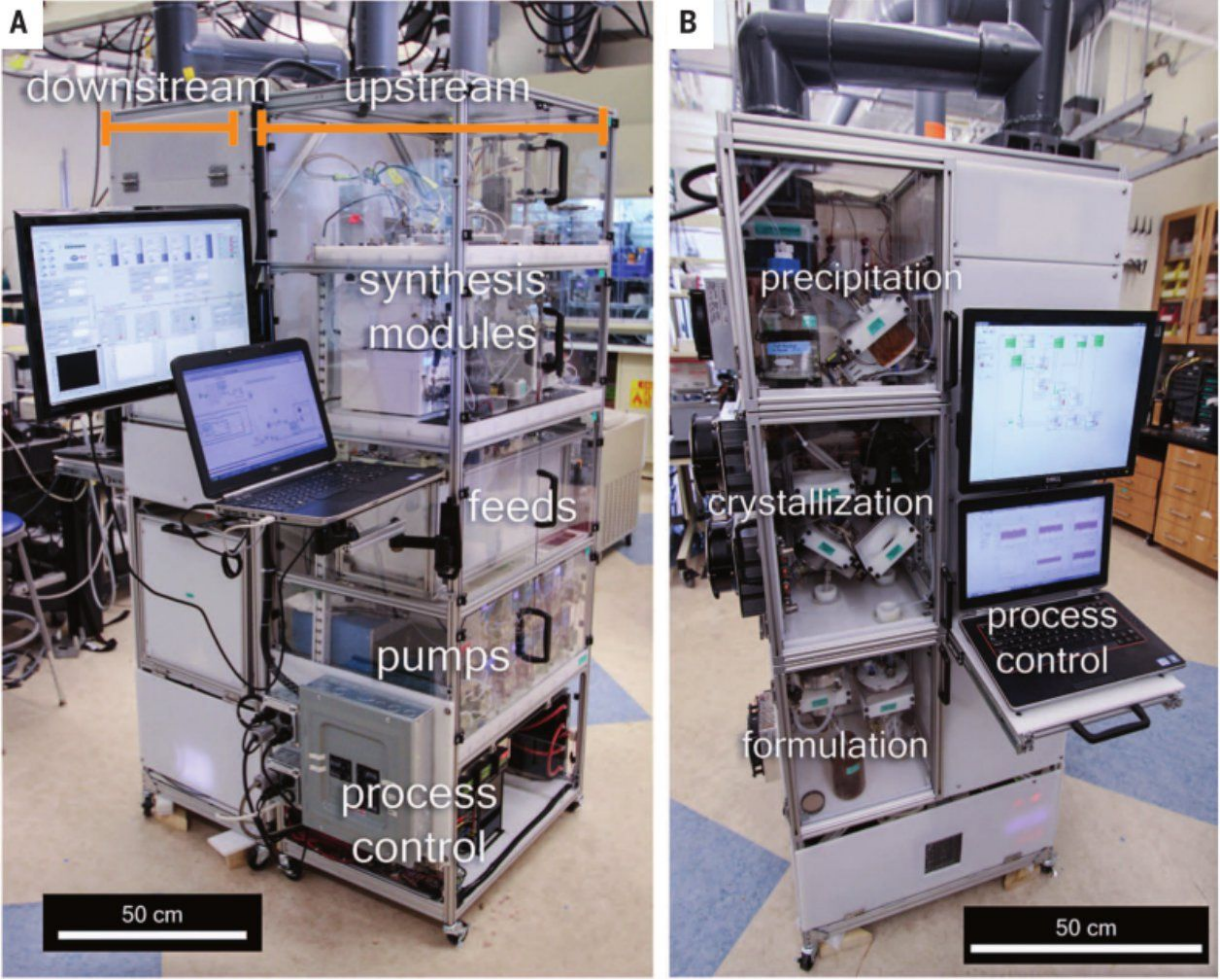
Reconfigurable system for continuous production and formulation of APIs. (Reproduced with permission from [85], copyright 2016 American Association for the Advancement of Science).

The future use of this kind of platform would concern the “on-demand” production or the “instantaneous” production of short-lived pharmaceuticals (Figure 4). Other advantageous concerns of this reconfigurable platform are the lower production costs, the higher safety, the automation (computer controlled processes), the reduced waste (production could be done where is needed and in the right amount).

## Conclusion

Flow chemistry and manufacturing engineering have become largely acknowledged as viable and very often superior alternative to batch processing. Continuous-flow techniques offer increased safety, scalability, reproducibility, automation, reduced waste and costs, and accessibility to a wide range of new chemical possibilities, seldom not accessible through classic batch chemistry. All those benefits are even more noteworthy and outstanding than what they might seem, because they widely fulfil most of the green chemistry principles. In this short overview, we tried to highlight progresses and potential of flow chemistry in the field of sustainable synthesis. Thus, it is expected that flow chemistry and microreactor technology could deeply change the way to perform sustainable chemical production in the near future [93].
